# Temporal Variation in Early‐Life Conditions Impacts on Later‐Life Levels of Infection in Sex Specific Ways

**DOI:** 10.1002/ece3.72132

**Published:** 2025-10-01

**Authors:** Hannah M. Ravenswater, Sarah J. Burthe, Thomas E. Reed, Mark A. Newell, Francis Daunt, Alice Carravieri, Ruth E. Dunn, Hanna H. V. Granroth‐Wilding, Carrie Gunn, Olivia Hicks, Emma J. A. Cunningham

**Affiliations:** ^1^ Institute of Evolutionary Biology, School of Biological Sciences University of Edinburgh Edinburgh UK; ^2^ UK Centre for Ecology and Hydrology Penicuik UK; ^3^ School of Biological, Earth and Environmental Sciences University College Cork Cork Ireland; ^4^ School of Environmental Sciences University of Liverpool Liverpool UK; ^5^ Lancaster Environment Centre Lancaster University Lancaster UK

**Keywords:** early life effects, macro‐parasites, parasitism, seabirds, seasonality, sex differences

## Abstract

Parasites are a fundamental component of wild animal populations, often inducing sub‐lethal chronic effects that impact host fitness and demography. However, the factors determining variation in infection burden are often poorly understood in wild systems. Environmental conditions can determine exposure to infection and the resources required to respond, but exhibit strong temporal variation. As environmental conditions are predicted to become more variable, it is crucial to understand how these conditions shape burden to predict the downstream effects on host populations. Early‐life conditions can shape responses to infection, potentially leading to delayed effects of environmental variation on fitness. The extent to which these are mediated by resources and later‐life conditions remains unclear and may vary between the sexes, who often differ in exposure risk and resource requirements. Here, we examine how differences in hatching and breeding conditions influence parasite burden throughout life. We utilise data from a long‐term population study of European shags (*Gulosus aristotelis*) on the Isle of May, Scotland, in which there is substantial variation in the timing of breeding within and between years, and nematode parasite burden can be measured in vivo using endoscopy. We show that adult parasite burden is influenced by seasonal and annual differences in current and early life conditions, but different patterns were observed in adult males and females. Burdens increased across the season in chicks and adult females but not in adult males. Instead, early life effects better explained burden in adult males, with those hatching later and in productive years displaying lower burdens. This suggests that early life may shape behaviour, immunity, or physiological development, impacting subsequent infection. Our findings reveal complex temporal effects on parasitism in species breeding in fluctuating environments. Incorporating seasonal and sex‐specific responses to parasitism is crucial to understanding how predicted environmental shifts could impact disease dynamics.

## Graphical abstract

Parasitism is a fundamental component of wild populations, but the factors that drive variation in the level of infection remain less clear. Here, we utilise a long‐term study population of seabirds to reveal how early life and current breeding conditions act differently between the sexes to impact natural variation in parasite burden. We find parasite burden increased across the breeding season in chicks and breeding females, but adult male burden showed a greater influence of early life conditions. Incorporating the impact of phenological variation and sex‐specific responses to parasitism is crucial if we are to understand how predicted environmental shifts could impact disease dynamics.

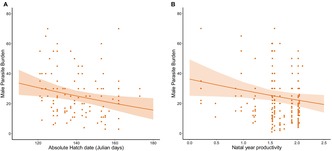

## 1 Introduction

Parasitism is a ubiquitous and fundamental component of animal populations influencing key demographic traits and ecosystem dynamics (May and Anderson 1983; Lafferty et al. 2008). Understanding how parasite burden in wildlife is determined is therefore key to quantifying population viability and the potential for parasite transmission between populations. The impact of pathogens on host populations is often modelled based on the survival outcomes of discrete infected versus uninfected states (Anderson and May 1979). However, many parasites induce sub‐lethal, chronic effects that impact individuals through resource depletion, as well as a range of key demographic traits such as reproduction and lifespan (Watson 2013). Furthermore, these effects are predicted to quantitatively increase as a function of parasite intensity (Anderson and May 1978). Whilst empirical associations between parasite burden and fitness‐related traits have been reported across a range of organisms (Ferreira et al. 2019; Hicks et al. 2019; Leivesley et al. 2019), the factors that fundamentally determine the underlying variation in parasite burden are complex and often poorly understood in wild systems. In free‐living animal populations, the combined influence of extrinsic factors such as climate and resource availability, with intrinsic factors such as physiological traits affecting immune defence, may determine both exposure and responses to infection (Sackett 2018). Untangling the ecological and environmental drivers of parasite burden is key to understanding the impact of infection in animal populations and of urgent importance given that variation in climatic drivers is predicted to increase in the future (Ummenhofer and Meehl 2017).

Environmental conditions affecting both host exposure to parasitism and their ability to deal with its costs can vary temporally within and across years. This variation may occur seasonally; therefore, we define seasonality as variability in timing within a year, which can be assessed across multiple years. Seasonal and annual variation in weather, resource availability and day length may affect the reproductive rate or survival of free‐living parasite stages (Tang 2009) or their vectors (Ewing et al. 2016; Akhmetzhanov et al. 2019), influencing the risk of host exposure to parasites. Such temporal variation can also be associated with the ability of hosts to raise an immune response (Xu et al. 2018; Nwaogu et al. 2019), thereby shaping resistance (Cornell et al. 2008; Arriero 2009) and tolerance mechanisms (McNew et al. 2019). Resistance involves the detection and removal of pathogens, aiming to decrease overall burden. In contrast, tolerance describes the ability of an individual to mitigate the negative fitness consequences of parasitism without altering the burden (Kutzer and Armitage 2016). Resource availability can play a crucial role in mediating these effects; however, in systems where transmission occurs through prey species, peak food abundance may also be associated with heightened exposure (Lafferty 1992). Host population composition may also vary seasonally, with individuals of different age, reproductive experience, or sex represented at differing levels in the population at any given time. These individual traits could shape the physiological capacity of individuals to respond to parasites both independently of, and through interactions with seasonality (Lynsdale et al. 2020; Valdebenito et al. 2021).

Seasonal and inter‐annual differences may also affect early life exposure to parasites in individuals born at different time points throughout a breeding season, with potentially long‐term consequences for parasite burdens later in life. High levels of exposure in juveniles may incur particularly high marginal costs of parasitism compared to adults due to the energetic costs of activities such as growth and developing thermoregulation (Garrido et al. 2016). On the other hand, higher parasite burdens in early life may prime the immune response, reducing subsequent infection (McDade et al. 2016). In contrast, lower parasite burdens may provide an environment in which individuals can invest in development without the diversion of resources to immunity, leading to good condition and the ability to resist or tolerate infection later in life. However, minimal exposure may reduce the benefits of immune priming, potentially leading to a higher burden in later life. Parental effects also influence immunity; the transfer of maternal antibodies may vary in relation to maternal responses to parasite exposure (Sparks et al. 2020), maternal condition (Coakley et al. 2014) and maternal resources availability (Ismail et al. 2013). In addition, juveniles may experience these changes in early life conditions indirectly, through differences in resource provisioning provided by parents (Granroth‐Wilding et al. 2015). These parental effects can subsequently influence the juveniles' long‐term responses to parasitism by influencing the development of adult immunity (Knutie et al. 2017), the adoption of behavioural strategies such as migration or foraging that subsequently influence exposure (Altizer et al. 2011) or survival into the adult population (Sparks et al. 2020). Hence, seasonal and annual variation in parasite exposure during development can have long‐lasting effects, depending on the impact on juveniles or their parents. Understanding how burden differences arise and are translated into adulthood is therefore key to deciphering the links between environmental conditions, parasitism and fitness.

Here we investigate the impact of seasonal and annual variation in early life conditions on parasite burden across an individual's lifetime, focusing on a nematode–seabird system in which there is substantial variation in the timing of host breeding across the season and in environmental conditions between seasons (Harris et al. 1994; Daunt et al. 1999; Keogan et al. 2020). Parasite burden has previously been shown to impact breeding success in females of this population (Hicks et al. 2019). In addition, early life conditions have been shown to influence fitness‐related responses to parasitism across the breeding season in experimental studies (Reed et al. 2012; Granroth‐Wilding et al. 2017). Specifically, 37% of the seasonal decline in breeding success is explained by parasitism in breeding females, which reduced maternal investment in male offspring (Reed et al. 2008), and the removal of parasites from parents increased survival in chicks hatching early but not late in the season (Granroth‐Wilding et al. 2015). However, the long‐term impact of seasonal early‐life effects on natural burden levels in adulthood remains untested. In this study, we quantify how differences in the timing of breeding within a season and environmental conditions between breeding seasons can influence nematode parasite burden in chicks and breeding adults. We then examine whether differences in the timing of breeding have consequences for parasite burden in adulthood. Our previous experimental work found that parasitism during breeding accounts for 37% of the observed seasonal decline in breeding success (Reed et al. 2008); we therefore predict that chicks hatched later in the season will have higher burdens consistent with the parasite‐induced reduction in breeding success in late breeding adults. For breeding adults, we expect one of two outcomes of early life conditions. Early life conditions associated with high burdens could be predicted to directly translate to high burdens later in life. Alternatively, a higher early life parasite burden may protect against later infection through the development of adaptive immunity, thus leading to lower burdens in later life.

## 2 Materials and Methods

### 2.1 Study System

This study was conducted in a population of European Shags (*Gulosus aristotelis*; hereafter shags) breeding on the Isle of May National Nature Reserve, south‐east Scotland (56°11′ N, 2°33′ W). This is a long‐term monitored population, with adults hatched in this dataset from 1994 onwards and parasite burden data collected between 2011 and 2019. Shags show substantial variation in hatching date and timing of the chick rearing period (Appendix S1) between and within years (Keogan et al. 2020). The species is sexually dimorphic, with males being 22% larger than females (Wanless and Harris 1997). Breeding starts at age 2 or 3 years, and both sexes provide parental care, carrying out incubation and provisioning offspring, with chicks fledging at 50 to 60 days (Cramp et al. 1983).

Since 1997, chicks have been ringed with uniquely coded colour rings, generating a breeding population of individually marked adults. Shags tend to return to the same areas to breed each year, allowing for resampling of individuals across their lifetime. Nest sites are routinely monitored using frequent, systematic checks throughout the breeding season (≥ 2 checks/week) in order to identify parents and record the presence of eggs and offspring. For this study, individuals were sexed as chicks by genotyping (Thanou et al. 2013) or as adults by vocalisations. Hatch date for all chicks in the brood was recorded either by direct observation during nest checks or back calculated based on chick wing length at ringing (around 20–30 days) (Daunt 2000). Hatch dates estimated using back calculation had an average difference of 1.6 ± 2.4 standard deviation (SD) days compared to direct observations (Grist et al. 2017), which is negligible given the substantial variation in hatch dates both within and between years (Appendix S1). In this study, individuals included in the adult data set hatched between 1994 and 2016, and chicks hatched in 2012, 2015 and 2019.

Shags feed benthically along coastal waters with a diet consisting mainly of fish and occasionally crustaceans (Howells et al. 2017). Shags are ubiquitously parasitized by nematode anisakid worms, which they ingest as larvae through their fish diet. Worms that reach maturity have been identified as *Contraceucum rudolphii*, whereas L3 larvae are potentially both *C. rudolphii* and *Anasakis simplex* (Unpublished data). The worms attach to the gut lining of the proventriculus. Prevalence is extremely high, being 100% in adults (Burthe et al. 2013) and 98% in chicks (Granroth‐Wilding et al. 2017). Parasitism is therefore an integral part of every individual's phenotype and fitness and can have substantial impacts on daily energy budgets, behaviour and breeding success; this has been shown both in relation to relative burden levels that occur naturally and experimentally through parasite removal experiments (Reed et al. 2008; Granroth‐Wilding et al. 2015; Hicks et al. 2018, 2019). Indices of population level exposure based on the number of nematode worms found in regurgitated food pellets suggest exposure increases across the breeding season (Appendix S2).

### 2.2 Quantifying Parasite Burden

Here we will use the term ‘burden’ to refer to the number of nematode parasites present within an individual host. Between 2011 and 2019, burdens of gastro‐intestinal anisakid nematodes were quantified in adult shags using live endoscopy under licence (see Burthe et al. 2013; Granroth‐Wilding et al. 2017). Endoscopy is a non‐destructive method allowing direct quantification of worm burdens in situ. This method is a more representative measure of worm burden than measures such as faecal egg counts (FECs) which are sensitive to worm density dependence and temporal variation in shedding (Ghosh et al. 2018). In this system, FEC‐based estimates of prevalence and burden were much lower than endoscopy‐based estimates, with some individuals having zero FECs despite endoscopy showing they were infected (Granroth‐Wilding et al. 2017). Chick nematode burdens were also quantified via endoscopy in three breeding seasons (2012, 2015 and 2019).

Endoscopy was carried out by a trained operator holding a personal licence operating under a project licence issued by the UK Home Office under the Animals (Scientific Procedures) Act 1986. Endoscopy has no known effect on either behavioural or fitness‐related traits (Burthe et al. 2013). Endoscopy was generally undertaken during late chick rearing (mean chick age of 17 ± 9.29 days). Previous studies (Burthe et al. 2013; Granroth‐Wilding et al. 2015) in 2011 and 2012 involved the treatment of individuals with anti‐helminthic drugs to investigate the direct and indirect effects of removing parasites in family groups. If a bird was treated, only endoscopies carried out before the date of treatment were included in the study. Control individuals that had been injected with saline were included in the adult data set as they show no difference in burden to non‐experimental individuals (Granroth‐Wilding et al. 2015). The majority of adults were sampled in multiple years, giving a total of 238 observations across 100 adult birds, 49 females and 51 males, in the 8 years of the study (Appendix S3). One measure of burden was obtained for the majority (176) of chicks, with a second observation undertaken later in the nestling stage for six individuals, giving 182 observations.

### 2.3 Measures of Within and Between Year Impacts on Parasite Burden

#### 2.3.1 Seasonal Effects Within a Year

To examine both the short term and long‐term effects of seasonal variation in conditions on parasite burden we explored the effects of hatch date in chick burdens, and hatch date and breeding date on adult burdens to consider both early life and current conditions. Breeding date was quantified as the timing of reproduction measured as the Julian hatch date of the oldest chick (Table 1). We considered two measures of hatch date: ‘absolute hatch date’ (Julian date of hatching from the 1st January) which measures a direct effect of breeding time on burden and ‘relative hatch date’ (date scaled to the median hatch date for that year) which measures how early or late an individual hatched relative to the population median that year (Table 1). Both measures of seasonality in early life were explored to assess the importance of timing relative to environmental fluctuations (absolute) and timing relative to conspecifics (relative). However, as these variables were correlated, they were not fitted in the same models.

**TABLE 1:** Explanatory variables included in models and the data set used for analyses.

Variable	Definition	Type of seasonal effect	Temporal scale	Data set	Appears in final global model
Absolute hatch date	Julian date of hatching	Early life	Within year	Adults and chicks	Adult
Relative hatch date	Julian date scaled to the season's median hatch date	Early life	Within year	Adults and chicks	Chick
Natal year productivity	The productivity in the year an individual hatched, used a proxy for environmental conditions. Productivity is calculated as the average number of chicks fledged per incubated nest in a series of undisturbed long term monitoring plots and fitted as a continuous variable	Early life	Between year	Adults and chicks	Adult and chick
Hatch year	The year in which an individual hatched	Early life	Between year	Adults and chicks	
Sampling date	Julian date when parasite quantification occurred	Early life	Within year	Adults	Adult
Breeding date	The timing of reproduction measured as the Julian hatch date of the oldest chick	Seasonal, sampling year	Within year	Adults	Adult
Sampling year productivity	The productivity (see above) in the year an adult is breeding	Seasonal, sampling year	Between year	Adults	Adult

#### 2.3.2 Environmental Effects Between Years

In addition to seasonal effects on parasitism, we investigated if differences in environmental conditions between years influenced parasite burden in chicks and adults. We considered two measures of productivity as a proxy for environmental conditions between years: ‘natal year productivity’ and ‘breeding year productivity’ (Table 1). The environmental factors leading to productive or non‐productive years are complex, multifactorial, and not fully understood and hence cannot be modelled directly. Productivity gives a relevant composite and quantitative measure of how good or bad breeding conditions were in any given year (Granroth‐Wilding et al. 2014; Hicks et al. 2019). Productivity is calculated as the average number of chicks fledged per incubated nest in a series of undisturbed long term monitoring plots, independent from endoscopy plots and fitted as a continuous variable. We also independently fitted ‘hatch year’, to account for natal cohort effects not explained by ‘natal year productivity’. Again, as these were highly correlated, they were not fitted together within the same adult models (Table 1).

### 2.4 Statistical Analysis

Model selection used an information theoretic approach, comparing a series of candidate models to identify the combination of drivers that best explained the observed variation in parasite burdens. Model selection was undertaken in the statistical programme ‘R’ (R Core Team 2018), by comparing Akaike information criterion (AIC) values which provide an estimate of the amount of Kullback–Leibler information lost by using a model to approximate full reality using the observed data (Johnson and Omland 2004), using the ‘AICmodavg’ package. The model with the lowest AIC value compared to alternative models was deemed the best supported. Models with an AIC within two of the best supported model were treated as equally supported unless they contained additional variables, in which case the most parsimonious modelled was favoured (Burnham and Anderson 2007). Model outputs were based on a mean centred values of continuous variables to aid the interpretation of coefficient estimates (Iacobucci et al. 2016). Global models were biologically informed based on pervious experimental and correlation studies in the system studies and only included interactions that were deemed biologically relevant. Initial exploratory modelling found better support for ‘relative hatch date’ compared to ‘absolute hatch date’ in chicks and ‘absolute hatch date’ compared to ‘relative hatch date’ in adults (Table 2). For between year effects, ‘natal year productivity’ was better supported than ‘hatch year’ for both chicks and adults (Table 2). Therefore, we present results for candidate models only including these better supported variables but complete model results including alternative variables are presented as Supporting Information (Tables S1.1 and S1.2). Quadratic effects of hatch date were included as candidate models in the analysis but as they did not differ in AIC to linear effects, they were not presented in the final results.

**TABLE 2:** The structure of the final global models used for the chick and adult data sets.

Data set	Final global model
Chicks	Burden ~ Relative hatch date + Natal year productivity + (1|BirdID) + (1|Endoscopy.Year) + (1|Age)
Adults (male and females analysed separately)	Burden ~ Absolute hatch date + Natal year productivity + Breeding date + Endoscopy date + Breeding year productivity + Chick age + (1|BirdID) + (1|Endoscopy.Year) + (1|Age)

#### 2.4.1 Modelling Seasonal Effects on Chick Burdens

The influence of the timing of early life on chick parasite burden was analysed using a Poisson distribution in generalised linear mixed models using the ‘lme4’ package (Bates et al. 2015). Model fit for different transformations and data distributions was compared using graphical inspection of model residuals for normality and homoscedasticity.

To examine the effect of differences in the timing of hatching on chick parasite burden in early life, we explored the effect of differences in hatch date on nematode parasite abundance using the variable ‘relative hatch date’, as a fixed effect (Table 2).

To investigate how variation in conditions between years influenced initial parasite burden we explored the influence of ‘natal year productivity’ (Granroth‐Wilding et al. 2014; Hicks et al. 2019), fitted as a fixed effect (Table 2). Chick age (in days) was included as a random intercept to account for variation in the age at which chicks were endoscoped. Bird ID was included as a random intercept to account for multiple parasite measures per individual. Nest was initially included as a nested random intercept with Bird ID to account for the presence of multiple chicks from the same nest, however as this did not significantly impact the AIC of models or the best supported model, this was dropped from the analysis. Sex was not available for all chicks; therefore, it was not included in the global analysis. Analysis for a subset of chicks with sex data is presented in Appendix S4 (Table A4).

#### 2.4.2 Modelling Current and Early Life Seasonal Effects on Adult Burden

Adult data were analysed using linear mixed models as these better fit the data distribution after square‐root transformation. Model fit for different transformations and data distributions was compared using graphical inspection of model residuals for normality and homoscedasticity. Males and females differ significantly in the distribution of parasite burden and in their hatching distribution across the season, with male burden consistently in a higher range than that of females and more male chicks hatching earlier in the season and in less productive years (Appendix S5). The impact of current and early life conditions on parasite burden was analysed separately, as the distribution of key variables differed, with adult males having both consistently higher burdens and male chicks hatching earlier in the season. Therefore, we analyse and present the sexes separately, in line with previously published analyses (Hicks et al. 2019). Although males and females from the same nest were present, this does not require accounting for due separation of the sexes in the analysis. However, the results of the analysis with the sexes pooled are presented in Appendix S7 (Table A7) for transparency.

##### 2.4.2.1 Current Seasonal Effects

To investigate the impact of current seasonal effects on adult parasite burden we included ‘breeding date’ as a fixed effect to represent seasonality in the year of sampling. Breeding date incorporates both extrinsic changes across the season with intrinsic differences between early and late breeding individuals (Table 1). Sampling did not always take place at the same stage of chick rearing, (20 birds on eggs, and 90 on chicks aged 17 ± 9.29 days), therefore we also included ‘sampling date’ as a fixed effect to account for general environmental variation with date. Brood age at sampling (age of the oldest offspring in the brood in days, or age of zero in the case of eggs) was included as a fixed effect in all models to account for variation in the phase of breeding at adult sampling for those adults in which chicks were hatched. In addition to examining seasonal effects on breeding adult burden, we also investigated the effect of between year differences in breeding conditions using ‘breeding year productivity’ as a fixed effect, derived in the same manner as ‘natal year productivity’ (Table 1). For all adult models we included endoscopy year, bird age (in years) and bird ID as random intercept. Age was included as a random intercept to account for variation in the age at which individuals were endoscoped. Bird ID was included to account for multiple parasite measures per individual.

##### 2.4.2.2 Early Life Effects

To examine the impact of the timing of early life on parasite burden in adulthood, we included ‘absolute hatch date’ to assess within breeding season effects and ‘natal year productivity’ as fixed effects to explore between year effects in our models. As in all adult models, endoscopy year, age and bird ID were accounted for in the random effects.

## 3 Results

### 3.1 Current Seasonal Effects on Chick Parasite Burden

**FIGURE 1:**
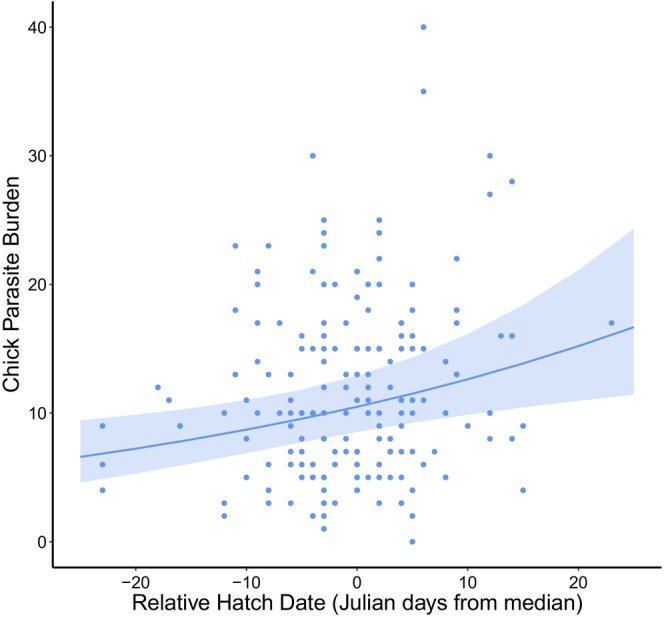
The relationship between chick parasite burden and relative hatch date in the Isle of May European shag population. Blue points represent the raw data with the predicted line plotted using the best supported candidate model. Shaded ribbons show 95% confidence internals and the plot was created using ‘ggplot2’ and ‘effects’ packages in R.

Parasite burdens in chicks increased with hatch date, such that relatively late‐hatched chicks had higher burdens than those hatched earlier in the season (Figure 1). This corresponds to, on average, a 55% increase in burden for every month past the season's median that a chick hatches. The best supported model included relative hatch date only (Table 3).

**TABLE 3:** The best fitting top five models for each adult data set and all models for the chick data set. Delta AICc and number of model parameters (*k*) is given for each candidate model and the effect size estimate (*E*) and standard error (SE) shown for the top ranked model in which fixed effects were supported, along with the null model. The best suported model is highlighted in bold. Outputs for adults are based on mixed effect models with a square‐root transformation and generalised mixed models for chicks; see written results for biologically meaningful interpretations.

Dataset	Model	Fixed effects	*E*	SE	*k*	ΔAIC
Chicks	**Burden ~ Scaled relative hatch date**	**Relative hatch date**	**0.019**	**0.006**	**4**	**0**
	Burden ~ Scaled relative hatch date + Natal year productivity				5	2.07
	Burden ~ Burden ~ 1				3	6.54
	Burden ~ Natal year productivity				4	8.58
Adult male models	**Burden ~ Absolute hatch date + Natal year productivity**	**Absolute hatch date**	**−0.026**	**0.011**	**8**	**0**
		**Natal year productivity**	**−0.650**	**0.306**		
	Burden ~ Absolute hatch date + Natal year productivity + Sampling date				9	1.75
	Burden ~ Absolute hatch date + Natal year productivity + Breeding date + Sampling date				10	1.87
	Burden ~ Absolute hatch date + Natal year productivity + Sampling year productivity				9	1.98
	Burden ~ Absolute hatch date				7	2.08
	Burden ~ 1				7	5.51
Adult females models	**Burden ~ Breeding date**	**Breeding date**	**0.037**	**0.010**	**7**	**0**
	Burden ~ Sampling date	Sampling date	0.037	0.010	7	0.48
	Burden ~ Absolute hatch date + Sampling date				8	1.35
	Burden ~ Absolute hatch date + Breeding date				8	2.02
	Burden ~ Sampling date + Breeding date				8	2.09
	Burden ~ 1				7	12.28

### 3.2 Current Seasonal Effects on Adult Burden

**FIGURE 2:**
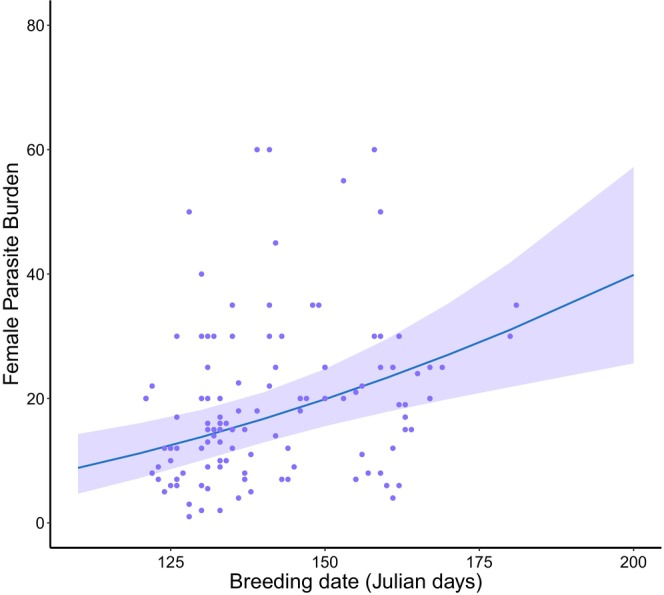
The relationship of breeding date with parasite burden in adult female European shags. Purple points represent the raw data with the predicted line plotted using the best supported candidate model. Shaded regions show 95% confidence intervals and the plot was created using ‘ggplot2’ and ‘effects’ packages in R.

We found that parasite burden increased across the breeding season in females (Figure 2) but not males. Both sampling and breeding date were equally supported for females, with females that were sampled and bred later in the season having higher burdens (an average increase of 7.25% for every month later breeding occurs). In contrast, breeding date was not present in the best supported models for adult males (Table 3). There was no effect of sampling year productivity on adult burden in either sex (Table 3).

### 3.3 Early Life Effects on Adult Parasite Burden

**FIGURE 3:**
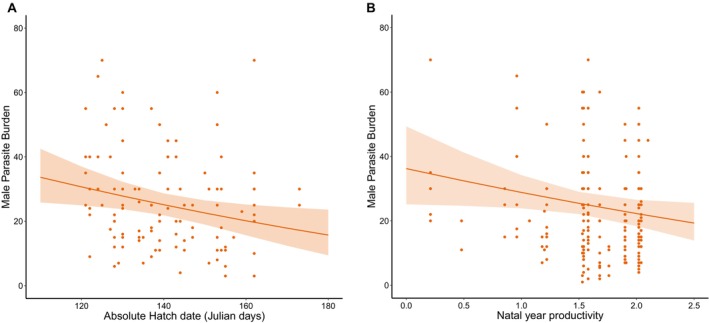
The relationship of absolute hatch date (A) and natal year productivity (B) with parasite burden in adult male European shags. Orange points represent the raw data with the predicted line plotted using the best supported candidate model. Shaded regions show 95% confidence intervals and the plot was created using ‘ggplot2’ and ‘effects’ packages in R.

Adult male parasite burdens were affected by both within and between year early life effects. These effects were again sex specific, but in contrast to the effects of breeding date, early life explained variation only in adult male burdens. Firstly, later‐hatched males have significantly lower parasite burdens as adults than those hatched earlier in the season (Table 3) (Figure 3A). In addition, our results revealed a male‐specific effect of natal year productivity (Table 3). Males hatching in more productive years had lower parasite burdens as adults, with an 11% decrease for every one chick increase in the average population‐level breeding success (Figure 3B). In contrast, neither hatch date nor natal year productivity appears in the model that best explained variation in adult female parasite burden (Table 3, Tables A3 and A4).

## 4 Discussion

In this study, we found that parasite burden in adult shags is influenced by both current and early life conditions, but the relative importance of these drivers is different in males and females. Our indices of exposure from food pellets suggest parasite exposure increases across the season. Parasite burden in chicks mirrored this pattern, with chick burden increasing across the season in both sexes. However, in adults, parasite load increased across the season in females but not males. Instead, in males, the effects of early life conditions were more apparent, with both temporal and productivity aspects of their natal year influencing their burden in adulthood. Males that hatched late in the season and in more productive years had lower parasite burdens as adults. Our results, therefore, reveal the importance of both seasonal and early life effects in shaping responses to infection, and that their relative importance may vary across life stages and operate differently in males and females.

### 4.1 Seasonal Effects on Chick and Adult Parasite Burden

Seasonality is a well‐established driver of variation in parasite burdens (Altizer et al. 2006; Albery et al. 2018; Shearer and Ezenwa 2020). However, our findings suggest that changes across the season are important in determining current burden in females and chicks but not males. This aligns with previous experimental studies in this system that found seasonal declines in breeding success were driven by parasitism impacting maternal but not paternal effects (Reed et al. 2008). Several mechanisms could drive an increase in parasite burden across the breeding season, categorised into factors affecting exposure or immune defence. Exposure may change across the season via variation in diet if different prey harbour different parasite loads (Leung and Koprivnikar 2019). In the Isle of May European shag population, chick diet switches as the season progresses (Howells et al. 2017), which may alter parasite exposure if prey consumed later in the season are more suitable intermediate hosts for developing worms. This is further supported by seasonal increases in parasite levels in shag pellets regurgitated following feeding (Appendix S2). In addition, in shags the sexes display contrasting foraging strategies, with females targeting a higher proportion of pelagic‐feeding prey (Lewis et al. 2015; Carravieri et al. 2020) which could also lead to sex differences in exposure.

Differences in parasite burden may also result from seasonal differences in the ability of individuals to deal with infection. Changes in immunity across the season in adult females could be driven by increasing costs associated with reproduction as the season progresses, or seasonally changing environmental cues, such as photoperiod, causing direct links between immunity and seasonality (Xu et al. 2018; Nwaogu et al. 2019). Seasonal changes in immunity in juveniles have been widely reported (Dubiec and Cicho 2005; Cornell et al. 2008; Arriero 2009). Differences in food quality and/or parental quality between early and late‐hatched individuals may also result in fewer resources available to mount and transfer immune protection. Immune differences in chicks may also be driven by effects on maternal antibody allocation as the season progresses, as offspring immunity is closely tied to maternal immunity at this developmental phase (Coakley et al. 2014). All these potential mechanisms of exposure, offspring and parent immunity, and maternal immune transfer are unlikely to act in isolation. Therefore, it is important to consider that effects could act simultaneously in opposing directions, creating complex outcomes for parasite burden.

### 4.2 Early Life Effects on Adult Parasite Burden

Early life conditions rather than current conditions better explain adult parasite burdens in males, with late hatched individuals and those hatched in more productive years having lower burdens as adults. This contrasts with the relationship observed in male chicks, with chick parasite burden increasing across the breeding season. This suggests that early life conditions and/or initial burdens may inform later life defence mechanisms and/or alter behaviours affecting subsequent exposure.

The decrease in parasite burden with hatch date observed in adult males may be a consequence of initial exposure if this primes the development of the immune system. High exposure to nematode worms of late hatch chicks may reduce the ability of infection to establish in adulthood through adaptive immunity. Models predict that the experience of infection can cause a reduction in susceptibility over time, but the level to which this occurs depends on the responsiveness of the immune system (Woolhouse 1992) and may be shaped by the ability of parasites to suppress host immune function (Zakeri et al. 2018). Differences in resource provisioning in early life may also affect allocation to the development of the immune system, with higher resource levels allowing greater immune investment and reduced burden in adulthood. For example, early life diet influenced the tolerance of parasite establishment in Cuban tree frogs ( *Osteopilus septentrionalis* ) (Knutie et al. 2017) and higher parental provisioning in years of high annual rainfall led to increased tolerance to parasitic flies in the Galapagos mockingbird ( *Mimus parvulus* ) (McNew et al. 2019). Although tolerance mechanisms do not necessarily relate directly to reductions in burden, experiencing early life conditions that enhance the ability of individuals to mitigate the fitness consequences of burden may reduce investment in costly defence, leading to differences in later‐life burdens. Previous work in European shags has suggested tolerance levels may differ between the sexes, as, despite males having higher burdens than females, burden has a greater effect on female behaviour and reproductive success than that of males (Hicks et al. 2019, 2018).

Alternatively, early life conditions may influence later‐life parasite burdens by determining behaviours influencing exposure to infection, such as foraging or migration. Later‐hatched chicks with higher burdens may go on to forage in different sites or forage less efficiently, leading to lower burdens in later life. Parasitism has been shown to increase total overwinter foraging time in adults (Granroth‐Wilding 2013); therefore, similar effects could be initiated by differences in early life burden. The Isle of May shag population is partially migratory, whereby approximately half of the population migrates to wintering sites primarily within northeastern Scotland during the non‐breeding season (Grist et al. 2017). The movement of individuals between habitats, sites, or populations may influence pathogen exposure and transmission (Gutierrez et al. 2017). Parasitism may also be a potential driver of migratory behaviour, allowing heavily parasitized individuals to escape or recover from high levels of parasitism in the breeding season environment (Shaw and Binning 2020). In shags, breeding date and migratory strategy are linked, with migratory parents breeding later than residents (Grist et al. 2017; Acker et al. 2021), potentially creating different levels of exposure in parents breeding at different times.

Interestingly, the effect of hatch date on adult burden was better explained by absolute hatch date, whereas in chicks relative hatch date was more important (Figures 1 and 3). Absolute hatch date integrates both differences in timing relative to environmental conditions, within and across years, capturing differences in the spread within a year but also differences in the timing across years (Keogan et al. 2020). The importance of inter‐annual differences in early life conditions in adults is further supported by the effect of natal year productivity on adult male burden (Figure 3).

### 4.3 Why Do We Observe Different Patterns in Males and Females?

The effect of early life conditions on later life success is often sex specific and generally greater in males (Wilkin and Sheldon 2009; Marshall et al. 2017) and parasite burdens are often higher in males than females (Zuk and McKean 1996), including nematode infections across 41 bird species (Valdebenito et al. 2020). This sex difference in burden is often attributed to the direct effects of differences in male and female sex hormones (Klein and Flanagan 2016). However, ecological factors may also contribute through sex differences in dietary exposure, physiological differences impacting resistance and tolerance, or differential mortality of one sex based on burden. In our population, males and females not only differ significantly in parasite burden (Hicks et al. 2018, 2019) but also in their hatching distribution across the season, with male burden consistently in a higher range than that of females and more male chicks hatched earlier in the season and in less productive years. This may shape the relative importance of seasonal effects for males and females from the outset.

In shags, males also require higher levels of food consumption, which is likely to be particularly marked in early life during the rapid growth phase (Daunt et al. 2001). Male chicks may therefore have higher exposure through their larger food intake. The resulting higher burdens in males may lead to sex differences in tolerance over an evolutionary timescale, possibly further exaggerating sex‐specific patterns of burden. This aligns with female shags paying a higher cost of parasitism than males, despite having lower burdens overall (Hicks et al. 2019). Differences in the timing of high energy requirements between the sexes may also lead to the costs of parasitism being established at different life stages. The male‐specific influence of natal year productivity on adult burden further supports the theory that parasitism in males is particularly sensitive to resource availability during development when males are potentially under greater constraint due to their higher requirements for growth.

The patterns we observe could also be affected by the mortality of highly parasitized, late‐hatched individuals. Late breeding parents are less likely to successfully rear costly sons, suggesting that there is a seasonal impact on male mortality at the chick stage (Reed et al. 2008), preliminary investigation of post‐fledging re‐sighting data suggests no evidence of sex‐specific mortality in our data set (Appendix S6). There remains a considerable amount of unexplained variation in our models, highlighting the complex and wide range of likely interacting factors that contribute to variation in parasite burden. Nonetheless, early life conditions play a role in shaping behavioural or physiological responses, influencing parasite burden into adulthood.

## 5 Conclusions and Wider Implications

This study demonstrates that parasite burden can be shaped by both past and current conditions that vary over time, but the patterns of these effects may be different in males and females. Climate change is predicted to lead to shifts in environmental conditions and phenology, altering the timing of both resource availability (Samplonius et al. 2020) and parasite exposure (Gethings et al. 2015) within the breeding season. In shags, the population mean lay date has advanced by 4 weeks over the last 30 years (Keogan et al. 2020), with a 24% decrease in the proportion of sand eels ( *Ammodytes marinus* ) in the diet of the population over a similar time frame (Howells et al. 2018). Marine parasite distributions are also potentially shifting with changing sea temperatures and host ranges (Byers 2021). Therefore, as the timing of breeding has a strong association with fitness and parasitism, there is the potential for environmental shifts to have complex effects on infection dynamics and population viability. Sex differences in the timing of seasonal effects could present challenges in predicting or managing parasite or disease outbreaks in populations. On the Isle of May shags, we may initially only see the impacts of phenological shifts in chicks and adult females, with delayed effects appearing when males reach adulthood. Understanding the relationships between breeding conditions, parasite exposure, and responses to infection will be key to predicting population viability and the dynamics of disease in this changing environment.

## Author Contributions

**Hannah M. Ravenswater:** conceptualization (equal), data curation (lead), formal analysis (lead), funding acquisition (equal), investigation (lead), methodology (equal), project administration (lead), visualization (lead), writing – original draft (lead), writing – review and editing (equal). **Sarah J. Burthe:** conceptualization (equal), data curation (supporting), formal analysis (supporting), investigation (supporting), methodology (equal), project administration (supporting), resources (supporting), supervision (equal), visualization (supporting), writing – original draft (supporting), writing – review and editing (equal). **Thomas E. Reed:** data curation (supporting), formal analysis (supporting), investigation (supporting), methodology (supporting), writing – review and editing (equal). **Mark A. Newell:** data curation (supporting), investigation (supporting), writing – review and editing (equal). **Francis Daunt:** data curation (supporting), investigation (supporting), resources (supporting), writing – review and editing (equal). **Hanna H. V. Granroth‐Wilding:** data curation (supporting), investigation (supporting), writing – review and editing (equal). **Alice Carravieri:** data curation (supporting), investigation (supporting), resources (supporting), writing – review and editing (equal). **Ruth E. Dunn:** data curation (supporting), investigation (supporting), writing – review and editing (equal). **Olivia Hicks:** data curation (supporting), investigation (supporting), writing – review and editing (equal). **Carrie Gunn:** data curation (supporting), investigation (supporting), writing – review and editing (equal). **Emma J. A. Cunningham:** conceptualization (equal), data curation (supporting), formal analysis (supporting), funding acquisition (equal), investigation (supporting), methodology (equal), project administration (supporting), resources (supporting), supervision (equal), visualization (supporting), writing – original draft (supporting), writing – review and editing (equal).

## Conflicts of Interest

The authors declare no conflicts of interest.

## Supporting information


**Appendix S1:** ece372132‐sup‐0001‐AppendixS1.docx.

## Data Availability

The data that support the findings of this study are made openly available in the Dryad data repository at https://doi.org/10.5061/dryad.pnvx0k70d.
