# Supplementary material for: Temporal Variation in Early‐Life Conditions Impacts on Later‐Life Levels of Infection in Sex Specific Ways
Source: Ecol Evol. 2025 Oct 1;15(10):e72132. doi: 10.1002/ece3.72132 (PMC12486190; doi:10.1002/ece3.72132)
Supplement: Supplementary file 1 — Appendix S1: ece372132‐sup‐0001‐AppendixS1.docx. [file ECE3-15-e72132-s001.docx]

**Appendix**

**Appendix 1**

***Between year variation in hatch date***

Chicks were sampled in 2012, 2015 and 2019. Individuals included in the adult data set hatched in the years: 1994, 1996, 1997, 1998, 2000, 2001, 2002, 2003, 2005, 2006, 2007, 2008, 2009, 2010, 2011, 2012, 2013, 2014, 2015, and 2016. These years showed extensive variation in the timing of breeding both within and between years (Figure A1).


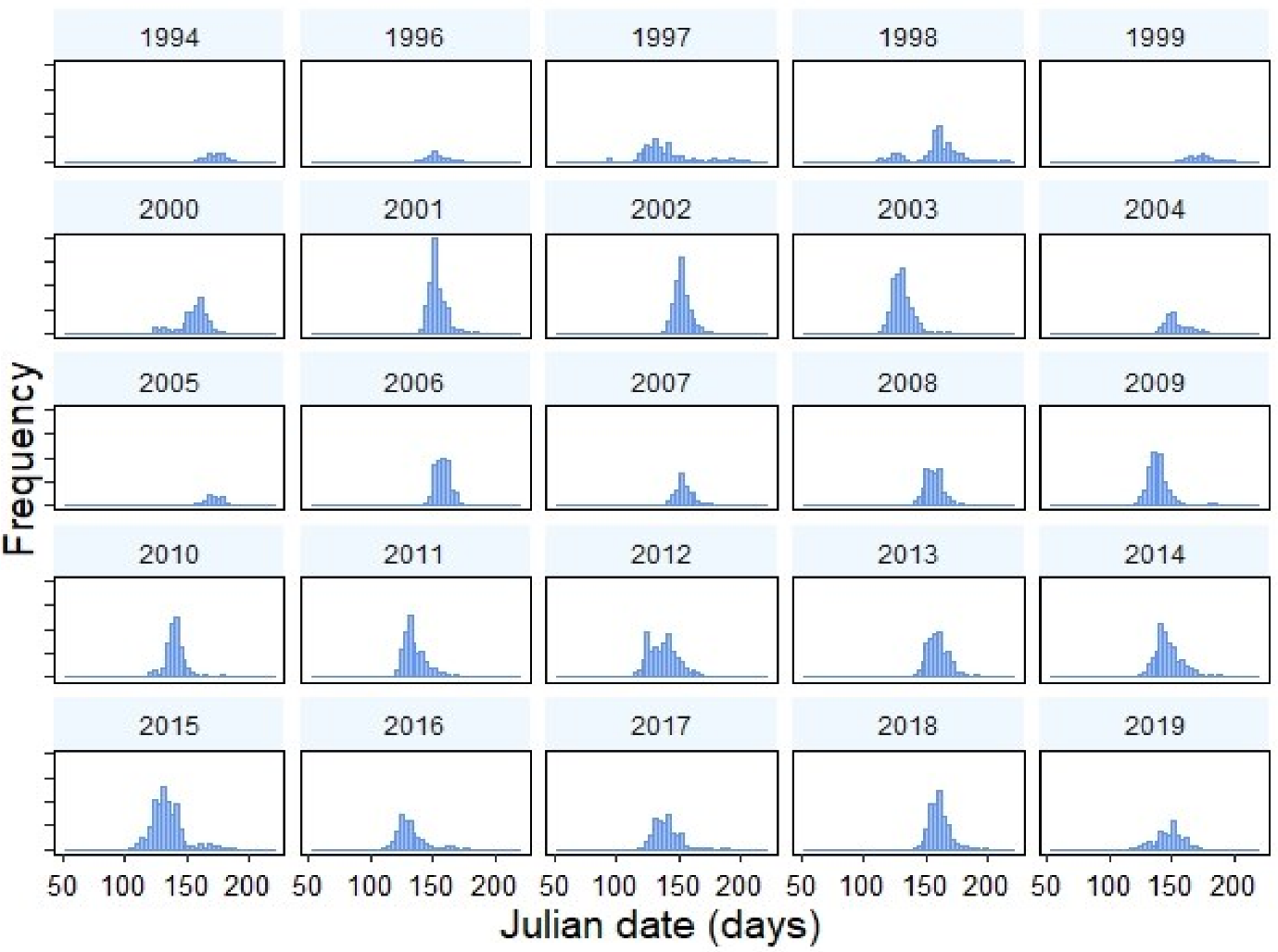


**Figure A1** The spread of hatch dates across the breeding seasons in the natal years included in this study for the European shag population on the Isle of May. This data was taken from a set of undisturbed long term monitoring plots, independent from endoscopy plots.

**Appendix 2**

**C*ould exposure be changing across the season?***

To obtain an index of parasite exposure levels across the season, regurgitated packages of undigested food produced by shags, known as pellets, were collected across the breeding season of 2005. The date of and areas (one of three areas) in which the pellets were collected was recorded, pellets were dissected and the number anisakid nematode worms present quantified. This data was analysed using generalised mixed models, with a Poisson distribution in R (R Core Team 2018), to investigate the effect of date of pellet collection on the total worm count of the pellet. The site of data collection accounted for as a fixed effect and an observation level random effect was used to account for over-dispersion. We found an increase in the count of anisakid nematodes in pellets produced across the breeding season of 2005 (GLMER, x^2^Z=56.1007.490, d.f=1, p<0.001) (Figure A2). This corresponds to a 30.5% increase in worm count found in pellets for every month the season progresses (~5 worms). As pellets cannot be assigned to a specific individual, they represent a population level proxy of exposure. There was no significant impact of collection site on the number of worms in pellets (GLMER, x^2^=1.420, d.f=3, p=0.701).


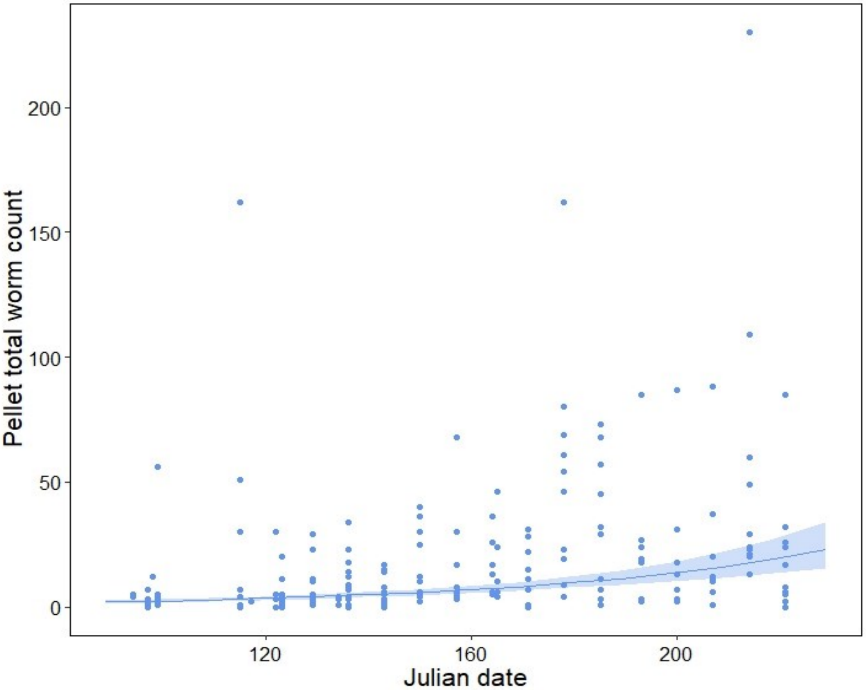


**Figure A2** The relationship between total worm count per pellet and Julian date across the season in the breeding season of 2005. A Julian date of 121 refers to the 1^st^ of May and 213 refers to the 1^st^ of August.

**Appendix 3**

***Breakdown of data by sex, life history stage and year***

 Individuals in the adult data sets were separate to the individuals used to determine the effect on chick burden. This lack of crossover was potentially caused by mass mortality events caused by extreme winter weather. In the adult data set males and females were sampled across all years (Table A.3).

**Table A.3** Break down of number of adult individuals sampled each year by sex.

**Appendix 4**

**Table A.4** Analysis of the subset of chick data for which sex was available. Due to limited power this was not included in the paper.

**Appendix 5**

***Differences in the distribution of early life variables between the sexes.***

To investigate if differences were present in their timing of hatching or the productivity in the individual's natal year, we fitted generalised linear mixed models, with a binomial distribution using the “lme4” package in the statistical programme “R” (R Core Team 2018). The effect of hatch date (absolute) and natal year productivity were tested on the sex of an individual and the brood size of the individual when it was ringed was also accounted for. An observation level random effect was used to account for overdispersion.

A significant effect of hatch date (GLMER, x^2^=3.901, d.f=1, p>0.05) and natal year productivity (GLMER, x^2^=14.056, d.f=1, p>0.001) was found on sex. A higher number of males were hatched earlier in the season and in less productive years.

**Appendix 6**

***Could early life conditions and differences influence chick mortality?***

To investigate whether either early-hatched or late-hatched chicks were disproportionately represented in the adult population, we fitted generalised linear mixed linear models using the “lme4” package in the statistical programme “R” (R Core Team 2018). Initially, to investigate whether late-hatched chicks may be disproportionately represented in the adult population, we tested the impact of hatch date and worm burden on the probability of a chick being re-sighted after the October in their natal year (therefore having survived into the winter). The effects of these factors were analysed for the entire chick data set, with the effect of sex analysed in the subset of the data set for which this information was available. Secondly, to investigate whether early-hatched chicks may be disproportionately represented in the adult population, we analysed the impact of hatch date and parasite burden on the probability of a chick being re-sighted any time after the October in their natal year (a crude proxy for survival). The same models but with the addition of sex were analysed for the subset of the chick data of known sex.

No effect of parasite burden (GLMER, x^2^=0.512, d.f=1, p=0.474) or hatch date (GLMER, x^2^=0.982, d.f=1, p=0.322) was found on the probability of re-sighting after October in their natal year. However the probability of re-sighting did differ significantly between hatch years (GLMER, x^2^= 10.77, d.f=1, p<0.01). For the subset of the chick data with sex available there was also no significant impact of sex on re-sighting probability (GLMER, x^2^= 0.0586, d.f=1, p= 0.809). Further, more advanced modelling is required to understand if these patterns are consistent during extreme weather events, as parasitized birds may be more vulnerable in these circumstances.

**Appendix 7**

***Results using a combined sex data set***

In addition to testing the sexes separately we also tested these effects in a combined sex data set of all adult individuals. Modelling was carried out using the same method as when sexes were analysed separately, but with the addition of candidate models containing two-way interactions between sex and early life or current breeding season variables.

1. ***Current seasonal effects on adult burden***

We found that parasite burden increased across the season with an average 9% increase for each week later sampling occurred. Seasonal increases during the breeding year also varied between males and females (best supported models contain sex * sampling date). Male burden slightly decreased with sampling date, whereas female burden increased through the season. Overall, male burden was significantly higher, by on average 15 worms, than females.

1. ***Early life effects on adult parasite burden***

We also found that over all adult parasite burden (sexes combined) was influenced by an individual’s absolute hatch date. Individuals that hatched later in their natal season had lower parasite burdens as adults with an average decrease of 7% every week later a bird hatched.

**Table A7** Top 10 models ranked by AICc for adults (sexes pooled) and null and sex only models. Delta AICc and number of model parameters (k) is given for each candidate model. Models are mixed models with a square-root transformation of the response variable. Random effects for adult models included; Bird ID, Year of endoscopy, Nest and adult age. The age of an adult’s offspring was accounted for as a fixed effect in all models.

**Impact**

**Supplementary information 1**

**Table S1.1.** Top 30 models ranked by AIC for adult males and adult females and null models. Delta AIC and number of model parameters (k) is given for each candidate model. Model outputs for adults are based a mixed model and on a square-root transformation. The age of an adult’s offspring was accounted for as a fixed effect in all models. Random effects for adult models included; Bird ID, Year of endoscopy, and age.

|  | **Model** | **k** | **Δ AIC** |
| --- | --- | --- | --- |
| *Adult male models* | Burden~ Absolute hatch date + Natal year productivity | 8 | 0 |
|  | Burden ~ Absolute hatch date + Natal year productivity + Sampling date | 9 | 1.75 |
|  | Burden ~ Absolute hatch date + Natal year productivity + Breeding date + Sampling date | 10 | 1.87 |
|  | Burden ~ Absolute hatch date + Natal year productivity + Sampling year productivity | 9 | 1.98 |
|  | Burden ~ Absolute hatch date | 7 | 2.08 |
|  | Burden ~ Absolute hatch date + Natal year productivity + Breeding date | 9 | 2.18 |
|  | Burden ~ Relative hatch date | 7 | 2.82 |
|  | Burden ~ Absolute hatch date + Breeding date + Sampling date | 9 | 3.24 |
|  | Burden ~ Absolute hatch date + Sampling date | 8 | 3.53 |
|  | Burden ~ Natal year productivity | 7 | 3.76 |
|  | Burden ~ Absolute hatch date + Natal year productivity + Breeding date + Sampling date + Sampling year productivity | 11 | 3.99 |
|  | Burden ~ Absolute hatch date + Sampling year productivity | 8 | 4.02 |
|  | Burden ~ Absolute hatch date + Natal year productivity + Sampling year productivity | 10 | 4.07 |
|  | Burden ~ Absolute hatch date + Breeding date | 8 | 4.09 |
|  | Burden ~ Relative hatch date + Breeding date + Sampling date | 9 | 4.14 |
|  | Burden ~ 1 | 7 | 5.51 |
| *Adult females*  *models* | Burden~ Breeding date | 7 | 0 |
|  | Burden ~ Sampling date | 7 | 0.48 |
|  | Burden ~ Absolute hatch date + Sampling date | 8 | 1.35 |
|  | Burden ~ Absolute hatch date + Breeding date | 8 | 2.02 |
|  | Burden ~ Sample date + Sampling year productivity | 8 | 2.09 |
|  | Burden ~ Breeding date + Sampling date | 8 | 2.28 |
|  | Burden ~ Relative hatch date + Sampling date | 8 | 2.29 |
|  | Burden ~ Natal year productivity + Sampling date | 8 | 2.29 |
|  | Burden ~ Breeding date + Sampling year productivity | 8 | 2.56 |
|  | Burden ~ Relative hatch date + Breeding date | 8 | 2.77 |
|  | Burden ~ Natal year productivity + Breeding date | 8 | 2.77 |
|  | Burden ~ Absolute hatch date + Natal year productivity + Sampling date | 9 | 3.61 |
|  | Burden ~ Absolute hatch date + Breeding date + Sampling date | 9 | 3.68 |
|  | Burden ~ Absolute hatch date + Natal year productivity + Breeding date | 9 | 4.31 |
|  | Burden ~ Breeding date + Sampling date + Sampling year productivity | 9 | 4.40 |
|  | Burden ~ 1 | 7 | 12.28 |

| *Dataset* | **Model** | **k** | **Δ AIC** |
| --- | --- | --- | --- |
| *Chicks*  *Total data set* | Burden ~ Scaled relative hatch date | 4 | 0 |
|  | Burden ~ Scaled relative hatch date + Natal year productivity | 5 | 2.07 |
|  | Burden ~ Absolute hatch date + Hatch year | 6 | 3.97 |
|  | Burden ~ Scaled relative hatch date + Hatch year | 6 | 3.97 |
|  | Burden ~ Absolute hatch date | 4 | 4.59 |
|  | Burden ~ Absolute hatch date + Natal year productivity | 5 | 5.26 |
|  | Burden ~ 1 | 3 | 6.54 |
|  | Burden ~ Natal year productivity | 4 | 8.58 |
|  | Burden ~ Hatch year | 5 | 10.70 |

**Table S1.2.** All models ranked by AIC for chicks. Delta AIC and number of model parameters (k) is given for each candidate model. Models are generalised mixed effect models for chicks with a Poisson distribution, with Bird ID and chick age (in days) as random effects.
